# Osteopathy

**Published:** 1910-10-08

**Authors:** 


*To the Editor of* The Hospital.

Dear Sir,—Would Mr. Barker kindly condescend to explain to us lay readers of The Hospital what he means by "bone-setting"? He writes that there are schools for teaching "bone-setting" in the United States with 12,000 students. Does he mean to infer that "osteopathy" and "bone-setting" are the same?

Dr. Wharton Hood was, I believe, most successful in treating sprains, tennis elbows, and cracked sinews, if that is the right diagnosis of the sudden pain and loss of use of leg which happens to cricketers and others generally when just starting to run. But his treatment was simply the very skilful application of strong adhesive strips of plaster, and then a "take up your bed and walk."

Does Mr. Barker profess to "set" a bone which (the usual story) has been not set by other surgeons; or does he profess to have some delicate touch which enables him to discover a muscle or ligament which has become displaced? If the latter, why call it *bone*-setting? Anyhow, will he tell us, and I ask in no carping spirit, what he does profess, and what bone-setters profess? It would add nothing to the importance of my questions were I to sign my name, and I refrain from doing so, as I do not want to be involved in any private correspondence.

Yours truly,

"A Mere Layman."

